# Unveiling the Truth: Fibroepithelial Polyp of the Vulva and Its Misdiagnosis as Cancer

**DOI:** 10.7759/cureus.61942

**Published:** 2024-06-08

**Authors:** Pankti U Tripathi, Prashant Suryarao, Meenal M Patvekar, Dipak Kolte

**Affiliations:** 1 Obstetrics and Gynaecology, Dr. D. Y. Patil Medical College, Hospital & Research Centre, Pune, IND

**Keywords:** vulval lesion, pedunculated growth of vulva, vulva, benign, fibroepithelial polyp

## Abstract

Fibroepithelial polyps (FEPs) are peculiar benign lesions that typically present as painless, pedunculated growths in various regions of the body, including the lower female genital tract. We discuss a case of a 45-year-old menopausal female who presented with an FEP in her vulva. The patient reported noticing a painless growth in her vulvar region for the past seven years, which had gradually increased in size. Clinical examination revealed a polypoidal, pedunculated, fleshy mass measuring approximately 11x8x7 cm in diameter. The lesion was excised under anesthesia, and histopathological examination confirmed the diagnosis of FEP. The patient had an uneventful postoperative course and showed no evidence of recurrence.

## Introduction

Fibroepithelial polyps (FEPs), commonly known as skin tags or acrochordons, are prevalent lesions that primarily affect adults, particularly obese women. Its incidence is 45% on average in the general population. They are usually small and found in the lower female genital tract, axilla, or neck [1]. Infrequently, they become large in size. Giant FEPs are extremely rare entities. While they are usually single, in rare cases, several lesions may appear. Young to middle-aged women frequently suffer from FEPs during their reproductive years. There is some evidence of hormonal association. Following partial excision, there is a chance of local recurrence. FEPs frequently exhibit a range of histological characteristics that vary widely, and they have a benign clinical course [2]. While FEPs of the vulva are uncommon, they can present diagnostic and management challenges due to their variable clinical presentation and resemblance to other vulvar lesions. Recurrence is rare and tends to occur only in cases of incomplete excision. We describe a case of FEP of the vulva and discuss its clinical features, diagnosis, and management.

## Case presentation

The patient was a 45-year-old, P3L3 postmenopausal woman who presented with multiple exophytic irregular-shaped polypoidal lesions with a warty appearance on the vulva, which had been increasing in size for the past seven years. Initially asymptomatic, they later rapidly expanded, causing discomfort during activities such as walking and sitting. She denied any history of trauma, bleeding, or discharge from the lesion. Her BMI was 24.1 kg/m^2^.

**Figure 1:**
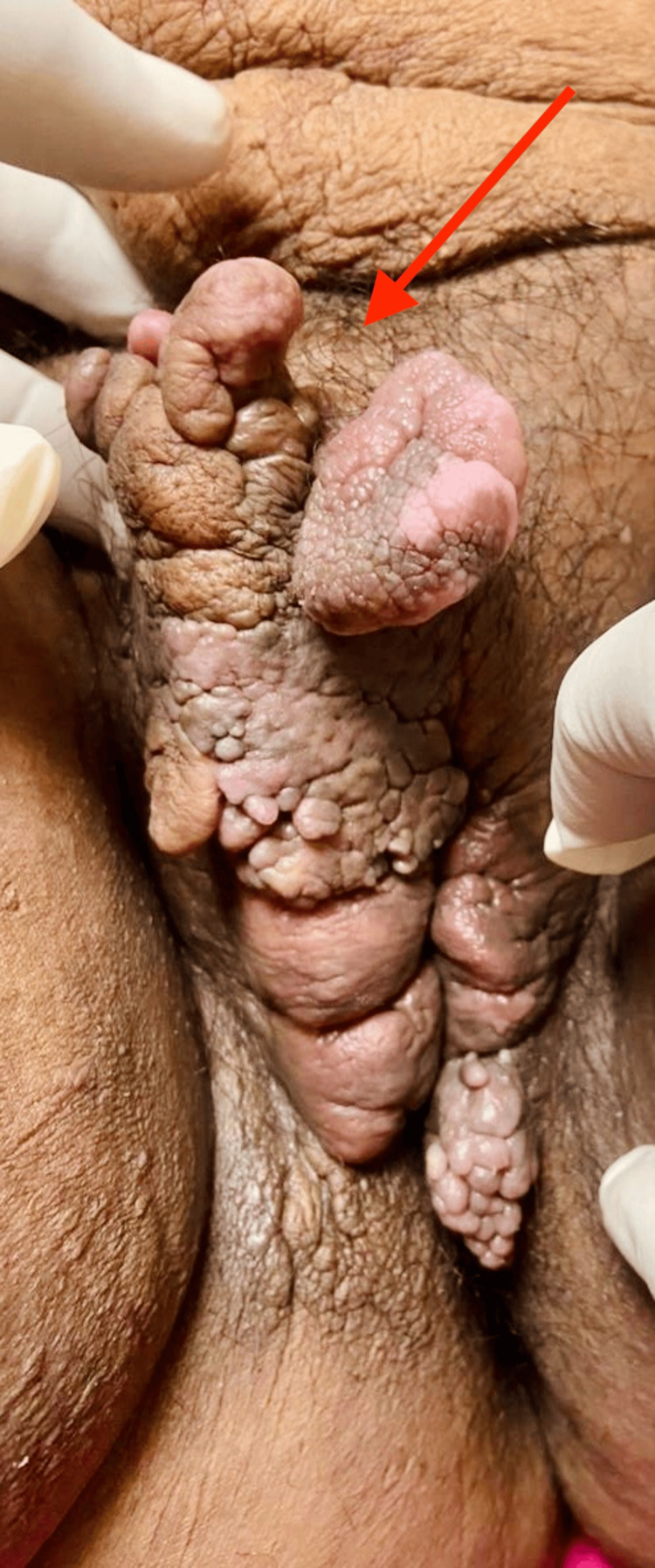
Irregular-shaped polypoidal lesion of the vulva

Physical examination was unremarkable, except for a large growth on the left and right labia, which extended up to the mons pubis. It was surrounded by erythema. The growth measured approximately 11x8x7 cm in size, and was firm in consistency, non-tender, and mobile (Figure 1). The lesion was non-pulsatile and non-reducible and did not increase in size with the Valsalva maneuver. It appeared well-circumscribed upon palpation.

**Figure 2:**
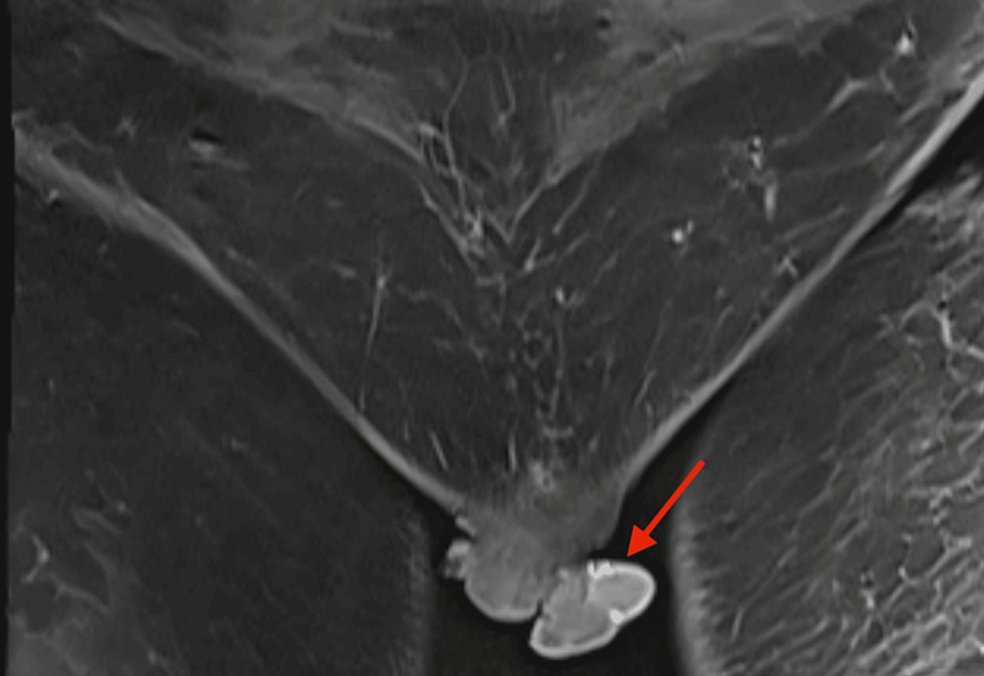
Coronal section of T1-weighted MRI of pelvis The image shows a large solid irregular mass with a polypoidal outline in the vulva involving both labia majora with exophytic extension MRI: magnetic resonance imaging

Blood and hormonal investigations were within normal limits, and HPV DNA was not detected. MRI of the pelvis indicated a large polypoidal mass in the vulva involving both labia majora with exophytic extension superiorly to involve the vagina up to the vault (Figure 2). It showed a polypoidal wall thickening of 14 mm. The fat plane between the lesion and the adjoining urethra and anterior wall of the anal canal were obscured, and the clitoris was not distinctly visualized. The mons pubis appeared normal. Bilateral inguinal lymphadenopathy was present, likely metastatic. Given the concerning appearance, radiological findings, and rapid growth of the lesion, suspicion of vulvar carcinoma was raised. A biopsy was recommended for further evaluation.

Multiple biopsies were performed and sent for histopathological examination, which were inconclusive. The patient and her relatives were thoroughly explained about the risks of vulvar carcinoma and its associated consequences. They consented to undergo an excisional biopsy with a frozen section under anesthesia. A team was prepared for radical vulvectomy with block inguinofemoral lymph node dissection given possible malignancy.

**Figure 3:**
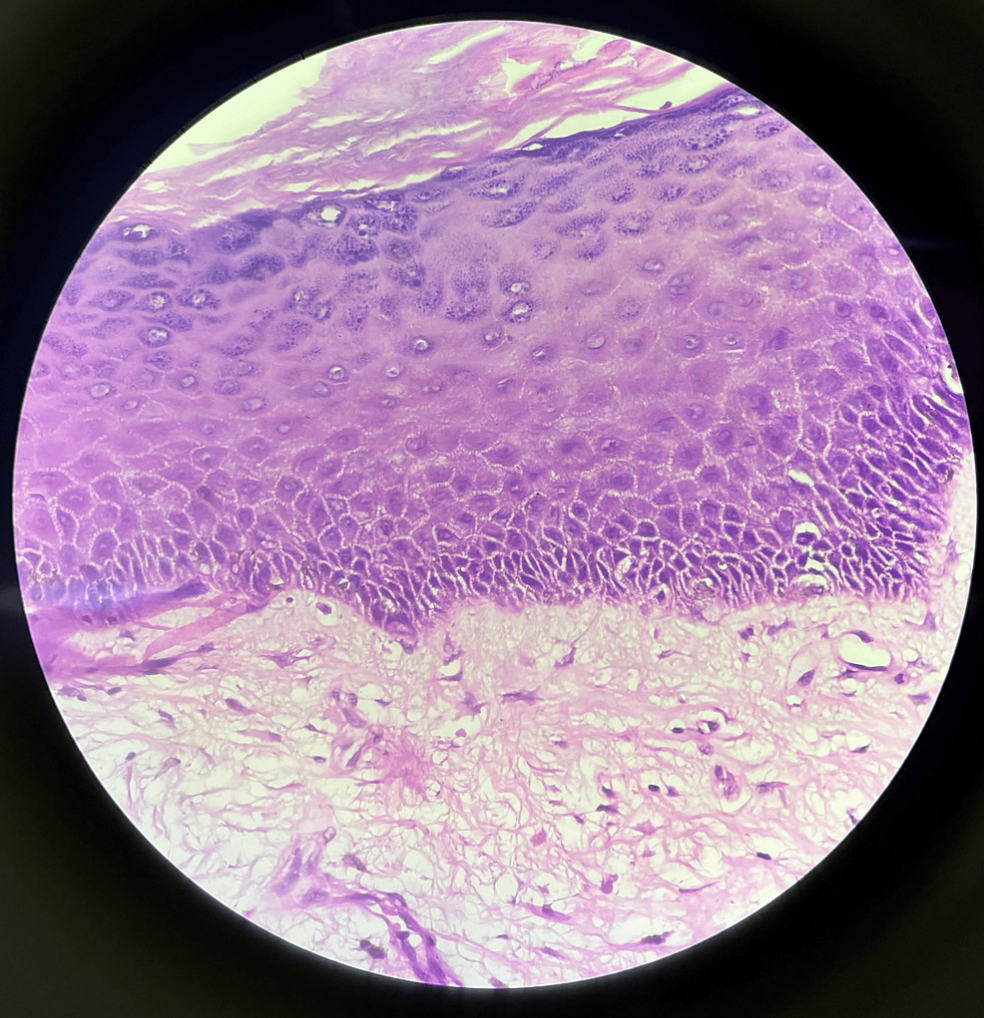
Hematoxylin and eosin (H&E)-stained image of the complete polyp under a 40x microscope The image shows a polypoidal structure lined by stratified squamous epithelium with underlying stroma, which is loose and contains elongated blood vessels with minimal lymphocytic infiltration around them

The lesion was completely excised with a small margin of normal surrounding tissue to ensure complete removal. Frozen section studies were negative for invasive malignancy, and hence, after adequate hemostasis, wound closure was performed. Histopathological examination revealed a polypoid lesion composed of fibrous stroma covered by stratified squamous epithelium with underlying stroma, which was loose and contained elongated blood vessels - suggestive of FEP (Figure 3).

The patient had an uneventful postoperative course and was discharged on postoperative day seven. She reported immediate relief of her symptoms and was satisfied with the aesthetic outcome of the procedure. At the one-month follow-up visit, the surgical site had healed well, with no evidence of recurrence.

## Discussion

The data on giant FEPs in the literature are limited to case reports due to their extreme rarity. These benign proliferations are often polypoid or pedunculated, rarely growing larger than 5 cm [3,4]. These polyps are hormone-sensitive and are predominantly found in women of reproductive age. They have also been reported in infants, pregnant, and postmenopausal women [5,6]. Giant FEPs of the vulva are rare but can cause significant morbidity in affected patients. Epithelial cells and fibrous tissue constitute these polyps. Their dimensions may vary, and they can be sessile or pedunculated [7].

Although FEPs are often painless, they can occasionally cause irritation or discomfort, particularly if they become larger or get inflamed from clothing or sexual activity-related friction. Though they are usually benign, any new growth or change in appearance in the vulvar region needs to be checked out by a medical expert to rule out more dangerous diseases like cancer. Benign FEP of the vulva can rarely resemble other vulvar growths, such as genital warts, cysts, and cancerous tumors. Histopathological evaluation is crucial for an appropriate diagnosis as clinical examination alone may not always be sufficient to distinguish these abnormalities. The treatment of choice is biopsy followed by wide surgical excision [8].

## Conclusions

While FEPs of the vulva are typically benign lesions, their clinical features and potentially alarming appearance may occasionally lead to misdiagnosis as vulvar carcinoma. Surgical excision is the preferred course of treatment for these polyps, leading to the resolution of symptoms and improvement in the quality of life. Normally, recurrence following excision is uncommon. It is imperative to keep an eye out for any recurrence or the emergence of new vulvar lesions, and long-term follow-up is advised to monitor for recurrence and ensure optimal outcomes.
